# Non-invasive cell classification using the Paint Raman Express Spectroscopy System (PRESS)

**DOI:** 10.1038/s41598-021-88056-3

**Published:** 2021-04-23

**Authors:** Yuka Akagi, Nobuhito Mori, Teruhisa Kawamura, Yuzo Takayama, Yasuyuki S. Kida

**Affiliations:** 1 Cellular and Molecular Biotechnology Research Institute, National Institute of Advanced Industrial Science and Technology (AIST), Central 5-41, 1-1-1 Higashi, Tsukuba, Ibaraki 305-8565 Japan; 2 Advanced Photonics and Biosensing Open Innovation Laboratory, National Institute of Advanced Industrial Science and Technology (AIST), Central 5-41, 1-1-1 Higashi, Tsukuba, Ibaraki 305-8565 Japan; 3 Tsukuba Life Science Innovation Program (T-LSI), School of Comprehensive Human Sciences, University of Tsukuba, 1-1-1 Tennoudai, Tsukuba, Ibaraki 305-8572 Japan; 4 Department of Biomedical Sciences, College of Life Sciences, Ritsumeikan University, 1-1-1 Noji-higashi, Kusatsu, Shiga 525-8577 Japan; 5 School of Integrative & Global Majors, University of Tsukuba, 1-1-1 Tennoudai, Tsukuba, Ibaraki 305-8572 Japan

**Keywords:** Raman spectroscopy, Classification and taxonomy, Machine learning, Lymphocytes

## Abstract

Raman scattering represents the distribution and abundance of intracellular molecules, including proteins and lipids, facilitating distinction between cellular states non-invasively and without staining. However, the scattered light obtained from cells is faint and cells have complex structures, making it difficult to obtain a Raman spectrum covering the entire cell in a short time using conventional methods. This also prevents efficient label-free cell classification. In the present study, we developed the Paint Raman Express Spectroscopy System, which uses two fast-rotating galvano mirrors to obtain spectra from a wide area of a cell. By using this system and applying machine learning, we were able to acquire broad spectra of a variety of human and mouse cell types, including pluripotent stem cells and confirmed that each cell type can be classified with high accuracy. Moreover, we classified different activation states of human T cells, despite their similar morphology. This system could be used for rapid and low-cost drug evaluation and quality management for drug screening in cell-based assays.

## Introduction

In Raman spectroscopy, molecular information is provided by Raman scattering of light emitted from a material irradiated with a laser beam of a certain frequency. The Raman spectrum is like the fingerprint of a material, containing chemical information such as the molecule type, chemical bonds, and structure. The measurement of Raman spectra does not require sample preparation, thus providing the advantage of non-destructive molecular analysis, unlike fluorescence staining and gene expression analysis. Since the first report of the use of Raman spectroscopy in cell biology to study *Drosophila* chromosomes^1^, it has been used for cells, tissue, and whole body studies^2,3^, and applied to a variety of biological validations, including the measurement of drug response^4^, evaluation of cell differentiation^5^, detection of cancer^6^, and analysis of cell death^7^.

There are two conventional methods of Raman spectrum measurement: point-scan^5,8–10^ and line-scan^11,12^. In point-scan Raman spectroscopy, a laser is focused on a single point on the sample, and the same objective lens is used to focus the Raman scattering light. However, since the scan region is only a few hundred nanometers in diameter, the obtained spectrum represents the information from a very limited area within the cell. To reduce measurement variations and evaluate a whole cell with complex structures, previous reports have shown that it is possible to either map the whole cell at a few micro intervals and obtain the average spectrum of a single cell^13^, or to classify cells by the average spectrum of multiple spectra obtained from random locations within the cell^14^. Using the mapping method, characteristic signals can be obtained in subcellular region or intracellular organelles, and it is also possible to obtain features suitable for cell classification. Conversely, in line-scan Raman spectroscopy, a cylindrical lens is placed in its optical path to focus the laser as a line on the sample^15,16^. The Raman scattering light at each spot on the line is simultaneously imaged on the slit of the spectrometer where it is incident and further detected by a two-dimensional charge-coupled device (CCD) camera. Since line-scan Raman spectroscopy allows for faster mapping of living cells than point-scan methods, it is often applied to the acquisition of Raman imaging of live cells^8,17–19^. Therefore, by mapping the whole area of a cell by line scan, the average spectrum of one cell can be obtained. It is also possible to extract spectra derived from specific cell organelles from the mapping data^20^. However, the acquisition of single cell spectral data using these conventional methods is time-consuming and can induce photothermal cellular damage by laser irradiation.

To overcome these problems, several methods have been reported to obtain Raman signals from large areas of cells in a restricted time. For example, by using a low Numerical Aperture (NA) objective lens, a large focal spot is created, and a large area of the cell is irradiated with the laser^21,22^. However, the use of low NA objectives results in low signal yield because the signal acquisition efficiency depends on the NA of the objective. Smith, N. et al. present *Hybrid Raman Imaging* that rapidly scans within a square region by a random scanning pattern within a pre-defined area^23^. Similarly, Schie et al. obtained the average Raman spectrum of a square region by moving the diffraction-limited spot, which they called the *Integrated Raman Spectra*^24^. Horiba's *Duo Scan Raman Imaging System* can scan a specific region in a raster scan fashion and obtain the average spectrum within the region^25^.

We developed the Paint Raman Express Spectroscopy System (PRESS), which uses two fast-rotating galvano mirrors, and can obtain the Raman spectrum of a wide cell area with high speed. Our system allows the user to specify a circular area, and the laser scans the specified circular area outside-in in a spiral fashion. This allows the laser to scan the circular region uniformly and acquire the regional spectrum in a short time. We believe that the short measurement time of the circular region is suitable for specialized measurements of floating cells such as T cells, cells in the process of passaging, and nuclear regions in cells. In this study, applying machine learning, using partial least squares (PLS) regression and support vector machines (SVM) to the spectral data obtained by PRESS, we classified cell types, including human pluripotent stem cells, and predicted the activation states of human T cells with high accuracy. Thus, the PRESS is suitable for single cell analysis, and by integrating artificial intelligence (AI)-based techniques we have demonstrated the ability to classify various cell types and activation states.

## Results

### Comparison of Raman spectra obtained by point and paint scanning

**Figure 1:**
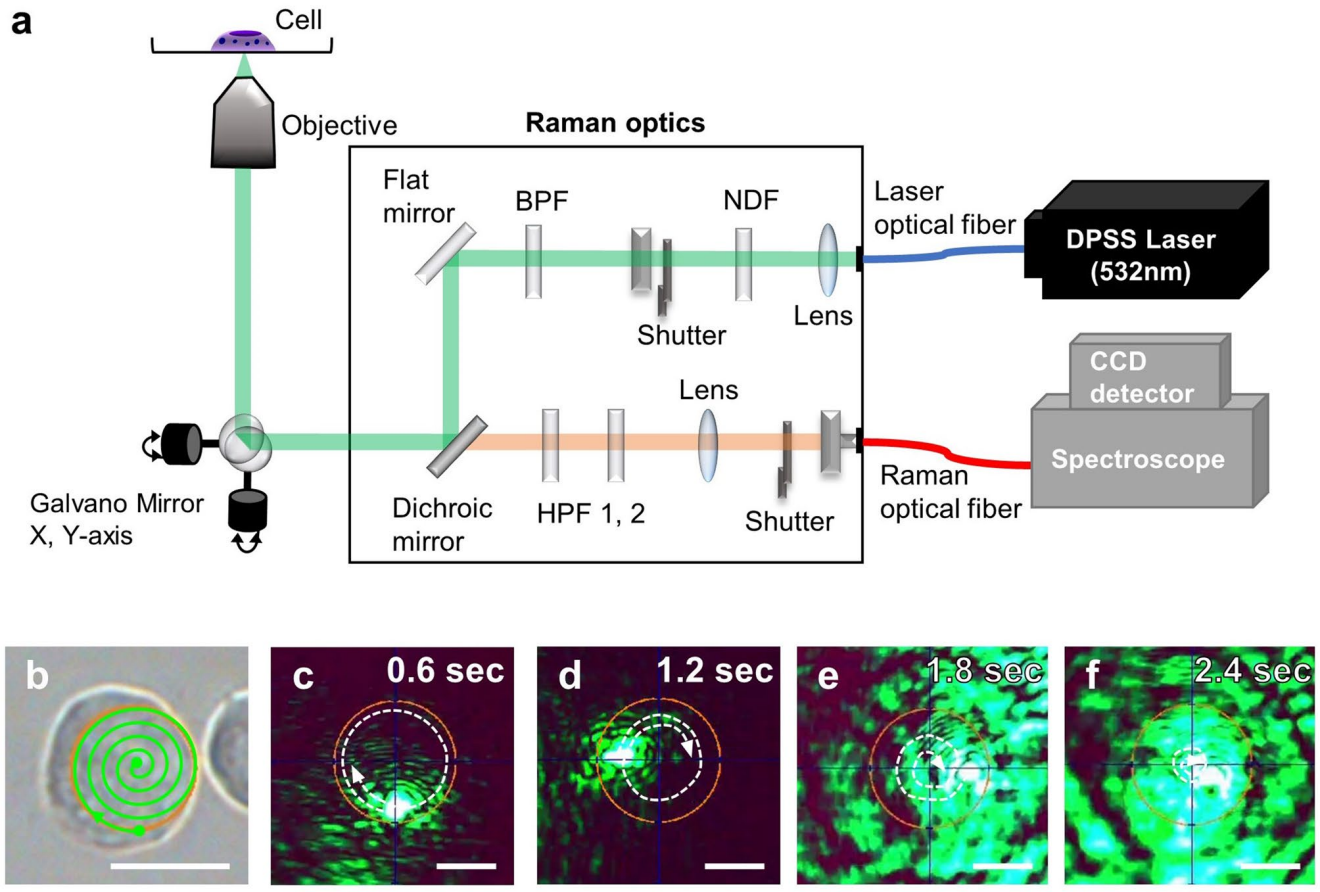
PRESS: A system to acquire a high-sensitivity Raman spectrum from a wide area in a single measurement. (**a**) Schematic of paint Raman spectroscopy system. Vibration of a dual-axis galvano-mirror enables moving the location of measurement for point excitation. (*NDF* neutral density filter, *BPF* band pass filter, *HPF* high pass filter). (**b**–**f**) Diagram of the laser beam path of the PRESS. (**b**) Laser path diagram of Jurkat cells in a circular area with 10 μm diameter irradiated by PRESS. Scale bar: 10 μm. (**c**–**f**) Selected time-lapse photos of the laser moving in a circular area of 20 μm diameter. The laser marking speed to irradiate the circular region in about 3 s was 1 mm ms^−1^. Scale bar: 10 μm.

To avoid site-associated variations in measurements due to the complex cellular structure, we developed the PRESS to obtain spectra from a wide area of the cell at high speed. By placing two rapidly rotating galvano mirrors in the optical path (Fig. 1a), it is possible to scan a specific area in a circular pattern (Fig. 1b, Supplementary Movie 1, Supplementary Fig. S1a). Since the rotation speed of the galvano mirrors can be controlled, it is possible to regulate the number of times the laser rotates in the area during the exposure time, enabling a uniform scan of the area (Fig. 1c–f). The measured region was distinguished from the brightfield image, and the shape (circle or square) and size (0.5–300 μm) of the region were adjusted. In the present study, we measured a circular region, especially since we used passaged cells in suspension for classification.

**Figure 2:**
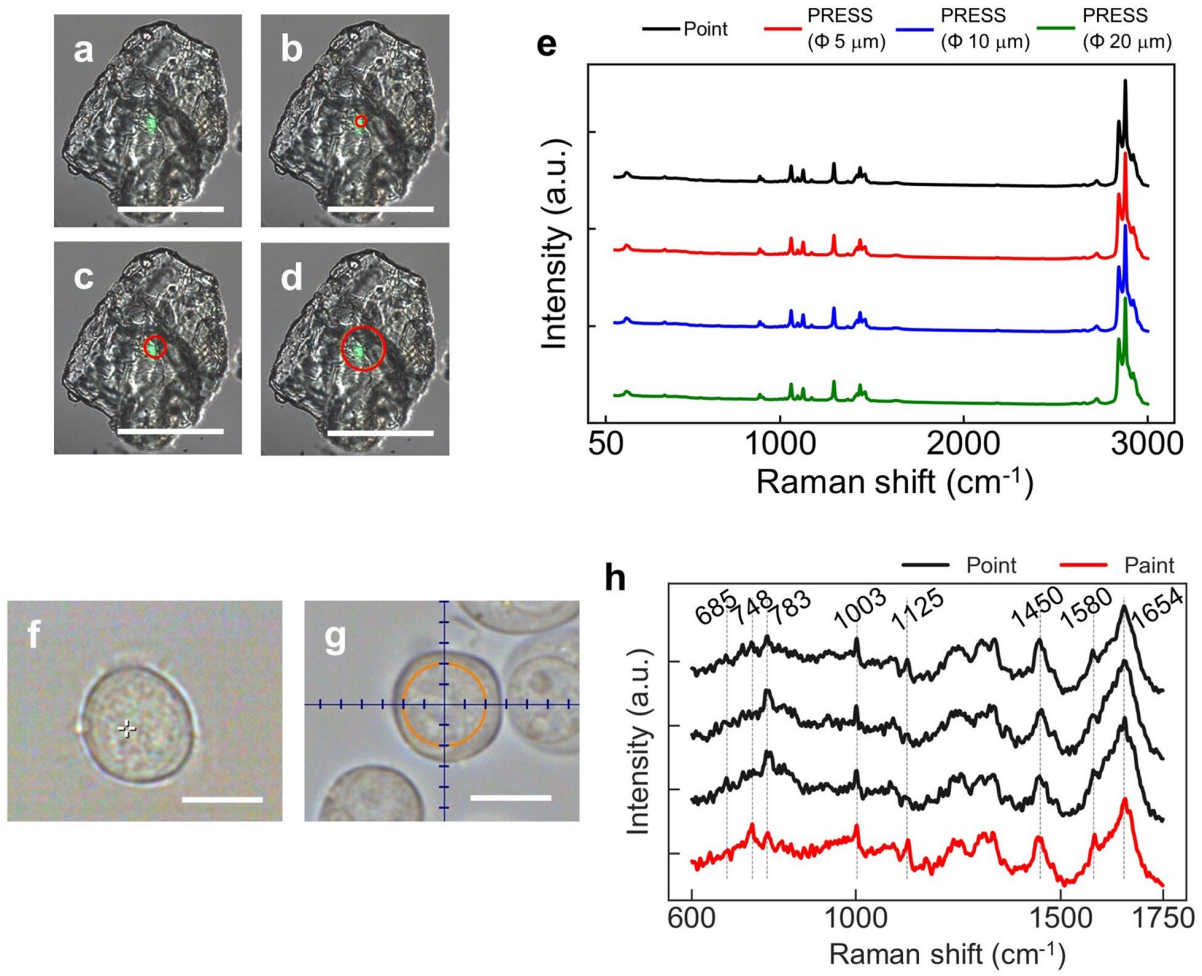
Comparison between Raman spectra by point and paint scanning. (**a**–**d**) Bright field image of solid palmitic acid. (**a**) Point-scan (laser spot diameter: < 450 nm) or (**b-d**) PRESS (circular areas of 5, 10, and 20 μm diameter: Φ 5, 10, and 20 μm). Scale bar: 50 μm. (**e**) Raman spectra detected by point-scan (black line), PRESS (Φ 5 μm: red line, Φ 10 μm: blue line, Φ 20 μm: green line). (**f**–**g**) Bright field image of Jurkat cells obtained by point-scan (**f**) or PRESS (**g**, Φ 10 μm). Scale bar: 10 μm. (**h**) Raman spectra detected by three point-scan (black line) or by PRESS (red line) from the same Jurkat cell. Some well-known biological peaks are shown: cytochrome C (748 and 1580 cm^−1^), nucleic acids (783 cm^−1^), proteins (1003, 1450 and 1654 cm^−1^), and lipids (1125 cm^−1^).

First, to confirm the utility of PRESS, we verified whether it could acquire detectable and valuable spectra, enough to classify the cell type, as the conventional point-scan method. The spectra of high-purity (> 95%) solid palmitic acid were obtained by both, point-scan (laser spot diameter < 450 nm) (Fig. 2a) and PRESS (Fig. 2b–d) methods, in circular areas of 5, 10, and 20 μm diameter within cells, and compared accordingly. The duration of exposure to the laser was set to 1 s for both methods, and the laser marking speed was adjusted such that the laser illuminated the whole area (1 mm ms^−1^), even in a circle with 20 μm diameter. We observed palmitic acid-derived peaks at 1059, 1124, 1293, 1419, 1435, 1462, 2842, 2879, and 2921 cm^−1^ in both methods (Fig. 2e). These spectra were checked against the spectral database KnowItAl^26^, and the palmitic acid spectra in the database (code: FFRX #478) matched those obtained by both methods (Supplementary Fig. S1b–d). Therefore, PRESS could produce spectral data comparable to that of the conventional point-scan method without loss of intensity.

Next, we validated the Raman measurements of living Jurkat cells using PRESS. The duration of exposure was set to 3 s, and the spectra of circular 10 μm diameter regions within the cells were obtained (Fig. 2g). Simultaneously, using the conventional point-scan method, three spectra were obtained at random locations with the same duration of exposure, i.e., 3 s (Fig. 2f). Both methods indicated characteristic peaks representing nucleic acids (685, 748, 783 cm^−1^), proteins (1003, 1450 cm^−1^), and lipids (1125, 1580, 1654 cm^−1^), in the fingerprint region 600–1800 cm^−1^ (Fig. 2h). The spectra obtained by PRESS did not show any decrease in sensitivity compared to those obtained by point-scan. In addition, the three point-scanned spectra showed some Raman shift regions with different intensities, such as 748 cm^−1^ and 1125 cm^−1^.

### Classification of different cell types using PRESS

**Figure 3:**
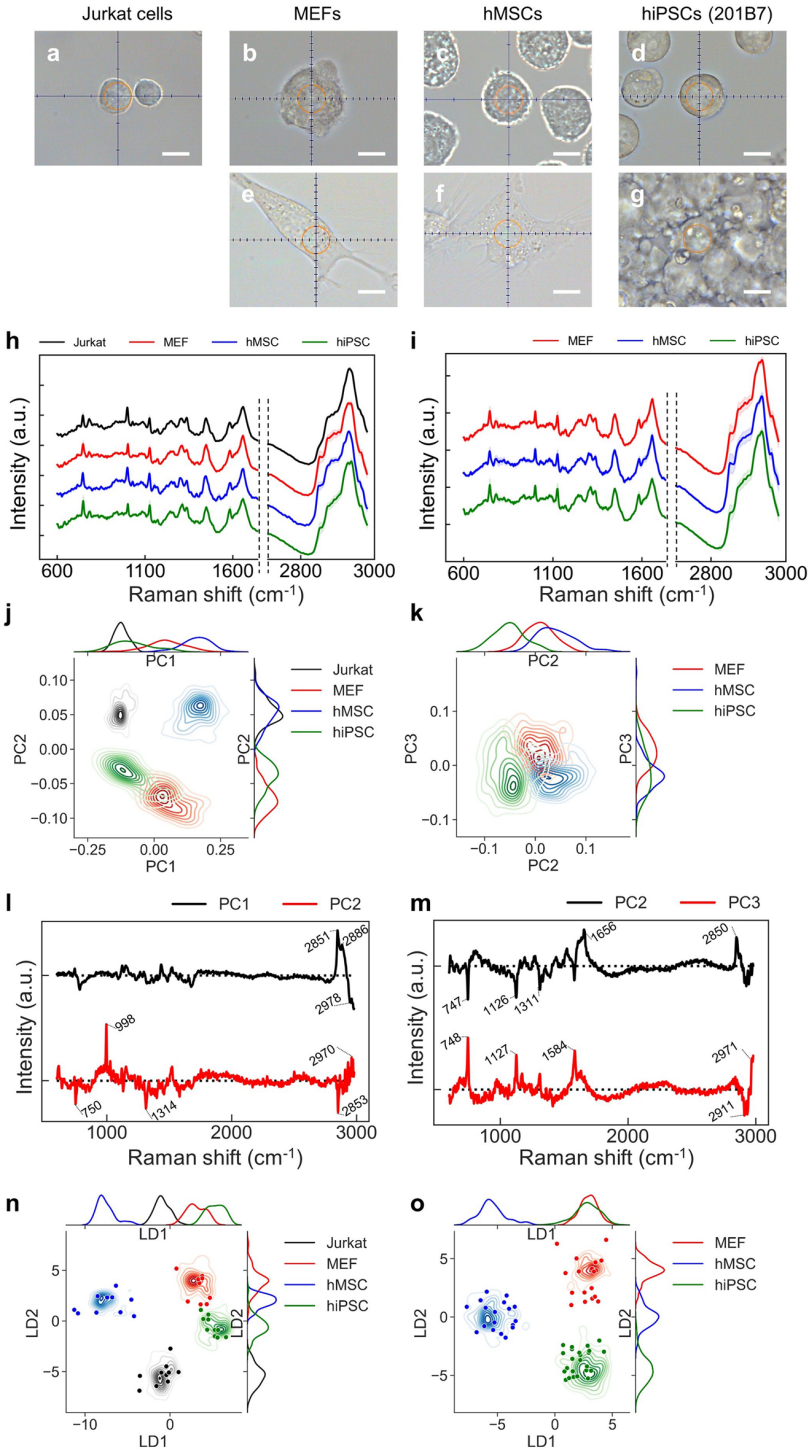
Classification of three or four typical cell types by PRESS. (**a**–**g**) Bright field image of Jurkat cells, MEFs, hMSCs, and hiPSCs. (**a**–**d**) Measurement of cells in suspension (a circular area of 10 μm diameter). (**e**–**g**) Measurement of adherent cells (a circular area of 10 μm diameter). Scale bar: 10 μm. (**h**, **i**) Average Raman spectrum measured by PRESS for each cell type (Jurkat: black, MEFs: red, hMSCs: blue, hiPSCs: green). SDs are shown by the shaded deviation. (**h**: cells in suspension, **i**: adherent cells). (**j**, **k**) Kernel density distribution of the PC axes for each cell type. On the x- and y-axes, each cell type distribution density is shown as lines. (**j**: cells in suspension (PC1, PC2), **k**: adherent cells (PC2, PC3)). (**l**, **m**) The loading vectors calculated by PCA. (**l**: cells in suspension (PC1, PC2), **m**: adherent cells (PC2, PC3)) (**n**, **o**) Kernel density distribution and scatter plot of LDA score along the first and second liner discriminant (LD1, LD2) axes for each cell type. Kernel density distribution shows training data, while scatter plot shows test data. On the x- and y-axes, the distribution density of each cell type in the training data for LD1 or LD2 is shown as lines. (**n**: cells in suspension, **o**: adherent cells).

To confirm whether the PRESS could be used to classify cell types, we obtained spectra from Jurkat cells, MEFs, hMSCs, and hiPSCs as representative cells. MEFs, hMSCs, and hiPSCs are usually cultured in an adherent state on culture dishes, whereas Jurkat cells are cultured in suspension. Thus, to match the culture condition of Jurkat cells, we used TrypLE or Accutase to obtain single cells from adherent cultures, and measured them in suspension (Fig. 3a–d). MEFs, hMSCs, and hiPSCs were also measured in the adherent state (Fig. 3e–g). Fifty cells in suspension were measured for each cell type (200 total), while 100 adherent cells were measured for each cell type (300 total). The obtained spectra were smoothed, baseline corrected, and normalized during pre-processing of the data. The averaged spectra data for each cell are shown in Fig. 3h,i.

To investigate the ability to differentiate between cell types, we used the obtained spectra as multivariate data for PCA using an unsupervised machine learning method. In the PCA, the top 100 of these PCs were extracted. Since the top five visually contributed to the distribution of cells, the distribution of cells against the five PCs is presented by a plot or kernel density estimation distribution as a pair plot (suspension measurements: Supplementary Fig. S2a, b; adhesion measurements: Supplementary Fig. S3). The contributions of the five PCs to dispersion of cells in suspension were 58.62%, 12.88%, 6.43%, 2.42%, and 1.76%, and in adherent cells were 49.72%, 12.29%, 7.16%, 3.93%, and 1.69%. The most effective visual classification between cell types in each paired plot diagram was achieved by PC1 and PC2 in suspended cells, and PC2 and PC3 in adherent cells (Fig. 3j,k, the loading vectors are shown in Fig. 3l,m). Jurkat cells, MEFs, hMSCs, and hiPSCs showed different cell distribution densities in both states. Especially in adhesive cells, PC2 contributed to the classification of hMSCs and hiPSCs, and PC3 contributed to the classification of MEFs and hMSCs. However, PCA-based dimensionality reduction did not contribute to a clear classification between all cell types, because the distribution of each cell type overlapped in some regions.

Therefore, we improved the classification of cell types by training a machine learning system with spectral data. We made use of LDA, a supervised learning technique that creates classification axes by extracting features between classes so that the separation of the classes is optimized. To verify the accuracy of the classification, all spectral data (200 cells in suspension and 300 adherent cells) were randomly divided such that 80% of spectra were training data and 20% were test data. Using the training data, LDA defined three or two new classification axes (the number of extraction axes was the number of classes minus 1) that best classified the cell types (Supplementary Fig. S2c). Then, we applied the test data to these extracted axes to verify the accuracy of the classification. Figure 3n,o show the distribution of cells against the LD1 and LD2 axes by cell type. The cells in the training data used to extract the classification axes were represented as kernel density distributions, and the cells used in the test data were plotted. The LDA classified the four or three cell types more accurately than the PCA, and the cells in the test data set were classified with 100% accuracy. Thus, LDA was able to classify three or four types of cells with high accuracy.

### Detection of different Raman signals from Jurkat cell states

**Figure 4:**
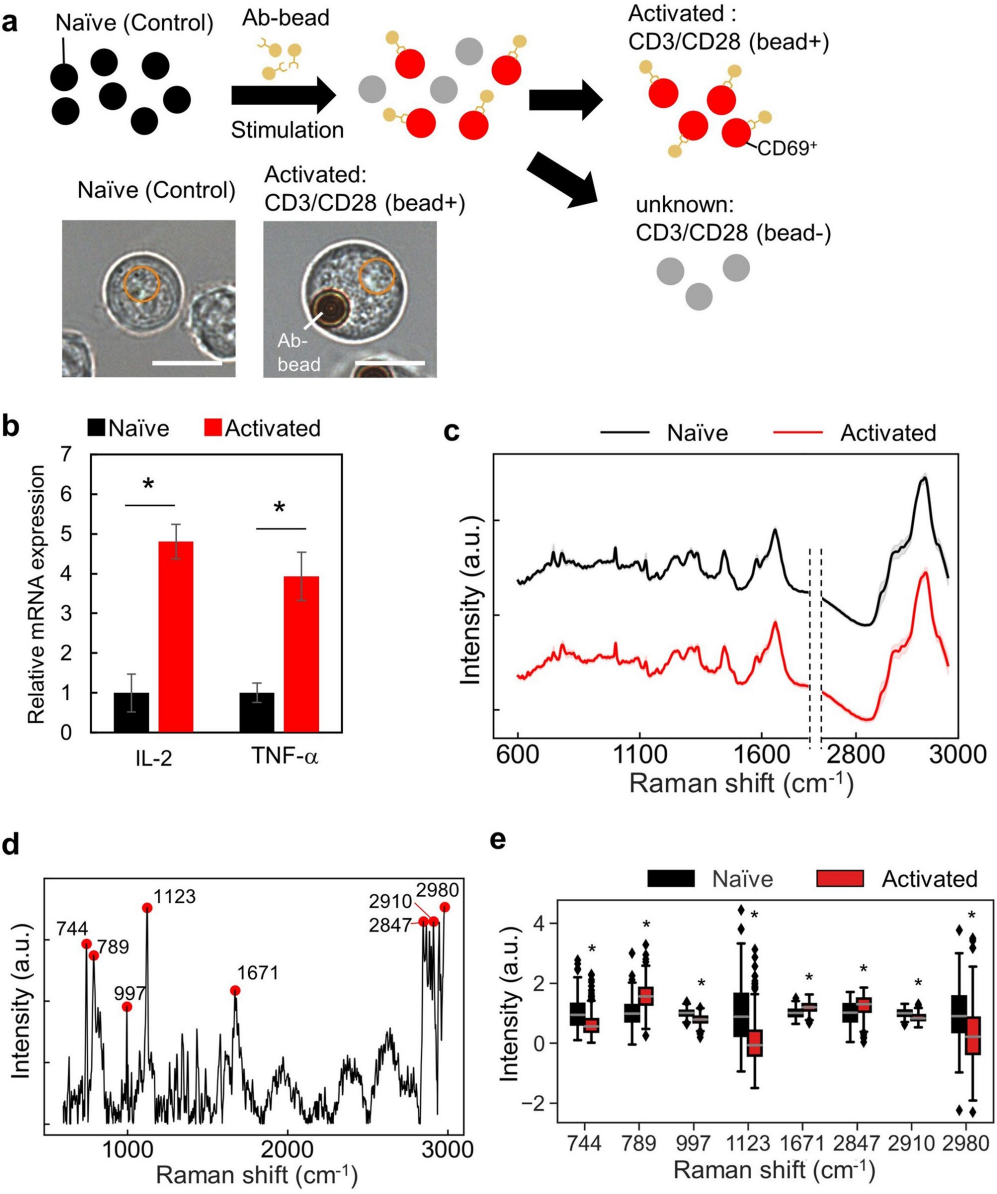
Comparison of Raman spectra of activated or naïve cells. (**a**) Schematic of cell stimulation of Jurkat cells using anti-CD3/CD28 conjugated beads. In the lower left, bright field image of activated or naïve cells is shown. The diameter of the circular area is 5 μm. Scale bar: 10 μm. (**b**) qRT-PCR of IL-2 and TNF-α mRNA from naïve or CD3/CD28-activated Jurkat cells (*n* = 3, error bar shows SDs; Student’s *t* test. *P < 0.05). (**c**) Averaged spectra of the analyzed region (600–3000 cm^−1^) for each cell type. SDs are shown by the shaded deviation. (black: naïve cells, red: activated cells). (**d**) Absolute value of spectrum intensity difference between control and stimulated cells. Detected peaks are shown as red plots (peak wave number: 744, 789, 1123, 1671, 2847, 2910, 2980 cm^−1^). (**e**) Comparisons of the spectrum intensity between naïve and activated cells for detected peak region. (n = 300; Error bars indicate the SD; median is shown as a grey line; Student’s *t* test or Welch's *t* test, *P < 0.05).

To further investigate the discrimination of different cell states in the same type of cells from the spectra obtained by PRESS, we stimulated the human T cell line, Jurkat, and classified activated and pre-stimulated naïve cells. In vivo, T cells, which serve as immune command posts, are activated by the recognition of antigens presented by antigen-presenting cells (APCs) by CD3-containing T cell receptor (TCR) complexes. Binding of CD28 on T cells and B7-1/B7-2 (CD80/CD86) on APCs is also involved in the activation of T cells. Once T cells are activated by the two signals, within about 2–4 h, the cell surface activation markers CD69, CD25, and HLA-DR are progressively upregulated, followed by increased secretion of cytokines such as IL-2 and IFN-γ. Many activated cells are CD69-positive at around 12–24 h after stimulation as the early activation phase and become CD25-positive for IL-2 receptor at 24–48 h as the mid-activation phase. In the late activation phase, after 48 h of stimulation, the number of HLA-DR-positive cells gradually increases^27^. In this study, we consider the cells after 24 h of stimulation, i.e., CD69-positive cells, as the activated cells and verify them. In this study, we used magnetic beads conjugated with CD3 and CD28 antibodies to stimulate T cells (Fig. 4a). First, we performed qPCR to confirm the activation and measured upregulated expression of the activation markers IL-2 and TNF-α in activated Jurkat cells compared to in naïve cells (Fig. 4b). We obtained 300 spectra from activated or naïve cells by PRESS. To avoid the influence of magnetic beads on the spectra, the diameter of the circular region was maintained at 5 μm. The average spectra of all cell types are shown in Fig. 4c. To verify the change in intensity of the spectra with activation, the peak difference between activated and naïve cells was calculated in absolute values (Fig. 4d). The significant peak regions detected by the *t*-tests are shown in red plots in Fig. 4d. We found that the activated and naïve cells had eight significantly different peaks between them (744, 789, 997, 1123, 1671, 2847, 2910, and 2980 cm^−1^) (Fig. 4d,e). The elements responsible for each peak are shown in Table 1^28^.

**Table 1:** Assignment of specific Raman bands to vibrational models and biological molecules^28^.

Peak (cm^−1^)	Assignment	Reference number
744 (746)	T (ring breathing mode of DNA/RNA bases)	^29^
789 (788)	O–P–O stretching DNA	^30^
997 (996)	C–O ribose, C–C	^31^
1123	(C–N), proteins (protein assignment) C–C stretching mode of lipids and protein C–N stretch, Glucose	^32^–^34^
1671 (1670)	Amide I, C = C stretching vibrations, Cholesterol & its esters C = C stretching vibration mode of steroid ring, Amide I (anti-parallel b-sheet) n(C = C) trans lipids, fatty acids	^35–^^38^
2847	CH_3_ symmetric stretch of lipids	^39^
2910	CH_3_ stretching vibrations	^40^
2980	CH, CH_2_, and CH_3_ symmetric and antisymmetric stretching of lipids	^41^

### Determination of the activation state of Jurkat cells using machine learning with spectral data

**Figure 5:**
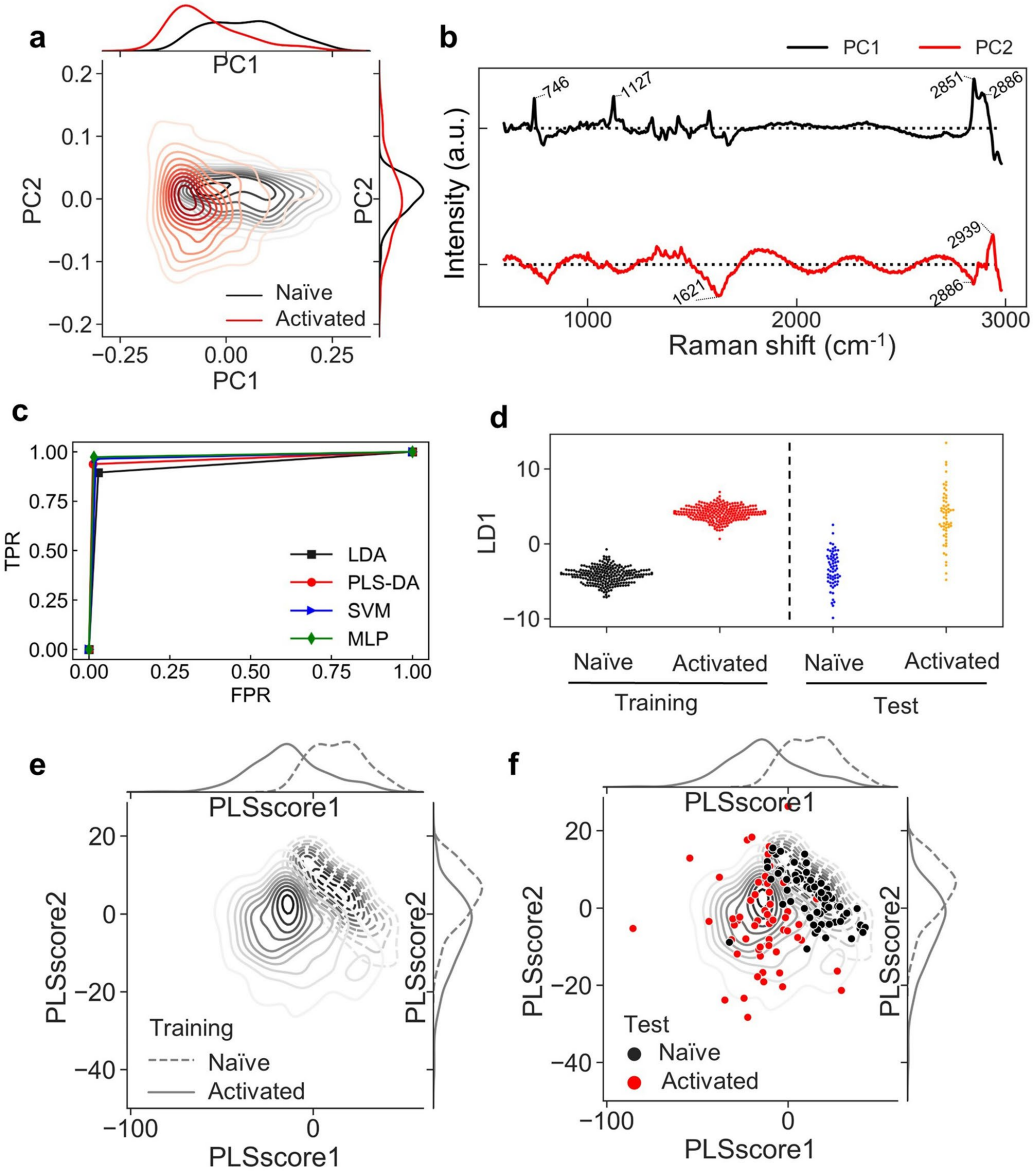
Classification of activated and naïve cells using machine learning. (**a**) Kernel density distribution plot of the first and second principal components for each cell type, which could clearly distinguish between two cell types. On the x- and y-axes, the cell distribution densities for PC1 and PC2 are shown as lines. (**b**) The first two loading vectors calculated by PCA. (**c**) Receiver operating characteristic (ROC) curves of classifiers (LDA, PLS-DA, SVM, and MLP). The X-axis denotes the false positive rate (FPR, 1-specificity), and the Y-axis represents the true positive rate (TFR). (**d**) Swarm plot of LDA score along the first discriminant axis. Each dot represents a single cell. The left side shows training data, and the right side shows test data. (**e**) Kernel density distribution diagram of the first and second PLS scores for the training data set of each cell type (naïve cell shown as dotted line, while activated cells shown as straight line). On the x- and y-axes, the cell distribution densities for PLS score1 and PLS score2 are shown as lines. (**f**) Test data is plotted above the kernel density distribution line of the first and second PLS scores of training data for each cell type (Training data: naïve cells are shown as dotted line, while activated cells are shown as straight line. Test data: naïve cells are shown in black, while activated cells are shown in red).

Characteristic peak changes were observed between activated and naïve cells. To perform dimensionality reduction and verify the classification, these spectra were used as multivariate data for machine learning. First, the spectral data in the Raman shift 600–2980 cm^−1^ region were applied to PCA, which extracted the top five PCs that contributed to the most variance. The contributions of the top five PCs were 43.7%, 10.1%, 7.8%, 4.5%, and 3.6%. The distribution of cells against the five extracted PCs were shown in pair-plots, and the loading vectors for each PC were also extracted (Supplementary Fig. S4). Of the five main components, the distribution of cells in PC1 and PC2 was slightly different from that of activated and naïve cells in the PC1 axis, although most of them overlapped and could not be clearly classified based on the PCA alone (Fig. 5a,b).

Therefore, we applied supervised machine learning to the spectral data and attempted to classify the activation state of Jurkat cells. We used LDA, PLS-DA, MLP, and SVM, which are machine learning techniques based on artificial neural networks, to explore methods that can classify activated and naïve cells with high accuracy. To perform SVM, we searched for a suitable parameter by the grid search method (linear, polynomial, radial basis function (RBF) kernel, sigmoid), regularization parameter (C), and kernel coefficient parameter (γ). Grid search revealed that the best parameters were C = 0.8500000000000002 and γ = 0.0001, and the best method was linear. For supervised learning, we first divided all data of activated and naïve cells randomly into training data (80%) and test data (20%). Using the training data, we extracted the axes to classify the two cell types based on four classification methods and classified the test data against these defined axes. Using the classified and original labels of the test data, FPR and TPR were calculated, and ROC curves were generated based on the FPR and TPR (Fig. 5c). In addition, we calculated sensitivity, specificity, RMSE, and the area under the ROC curve (AUC), which indicates the classification accuracy (Table 2).

**Table 2:** Validation of a classifier using a supervised machine learning method for the classification of activation states.

Method	Sensitivity	Specificity	AUC	RMSE
LDA	0.90	0.97	0.93	0.25
PLS-DA	0.94	0.99	0.96	0.18
SVM	0.96	0.98	0.97	0.16
MLP	0.97	0.98	0.98	0.13

To avoid the bias of division between training data and test data, we repeated the classification test 10 times, and the average values are shown in Table 2. The results showed that the accuracy (AUC) of DA, PLS-DA, SVM, and MLP were all high at 0.93, 0.96, 0.97, and 0.98, respectively (max: 1.00). The classification results of the training and test data were validated using LDA and PLS-DA, which were also used as a dimensionality reduction method (Fig. 5d–f). In PLS-DA, five features that contributed to the classification were extracted as PLS scores (Supplementary Fig. S5). The two-dimensional distribution images with scores1 and 2 showed the most visual classification of activated and naïve cells (training data: Fig. 5e, test data: Fig. 5f). Thus, the supervised learning method enabled us to classify activated and naïve cells with high accuracy.

### Prediction of Jurkat cell state by PRESS and machine learning

Finally, we examined whether cell state could be predicted using the spectra measured by PRESS. We used CD3/CD28 antibody beads to stimulate cells with unknown state, to which the beads were not bound. The unknown cells were expected to be either naïve cells that had not yet bound to the antibody-beads or activated cells, the beads of which had diverged after stimulation. However, to know the activation status, staining via conventional biological methods was required (Fig. 4a).

**Figure 6:**
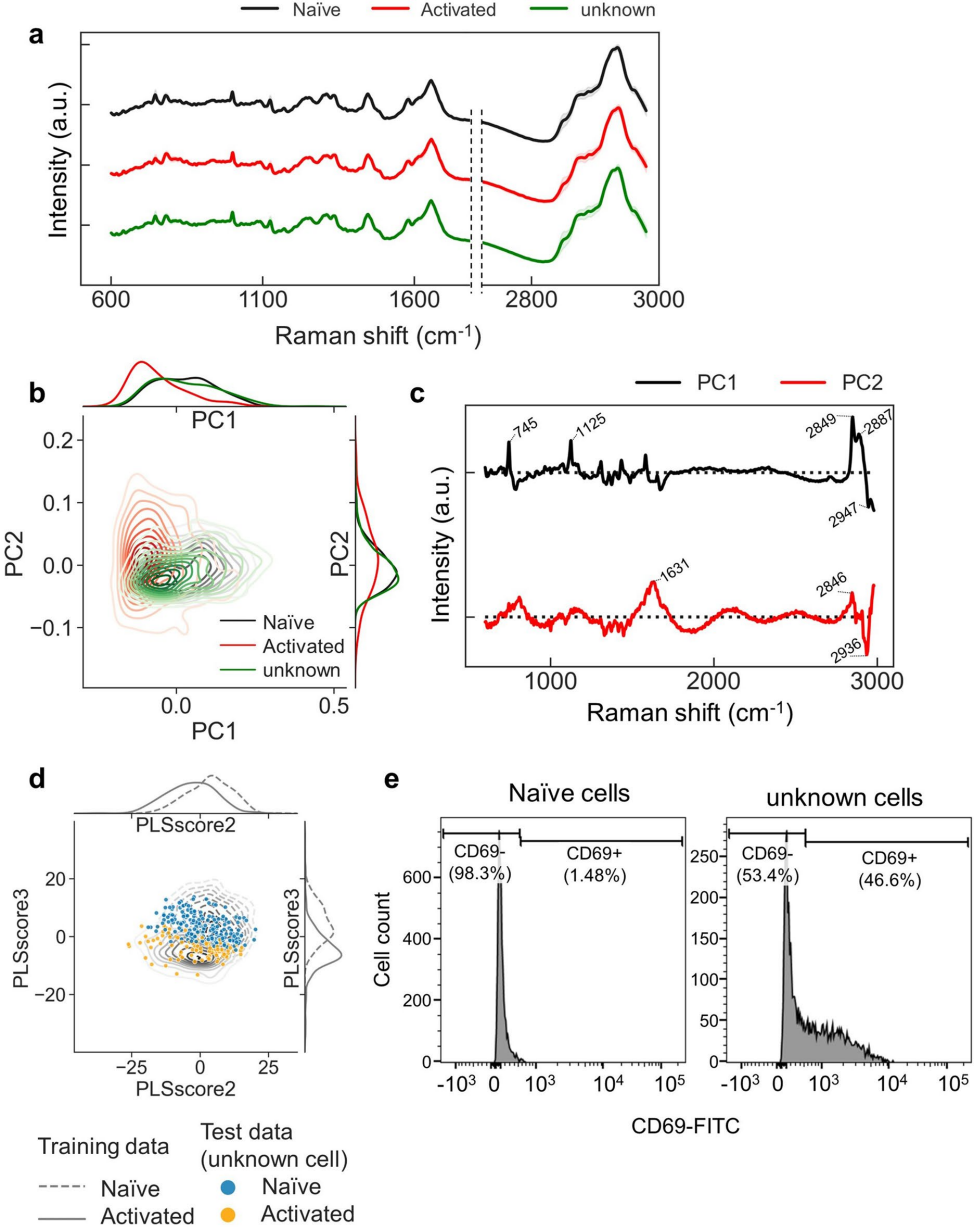
Prediction of unknown state of Jurkat cells using machine learning. (**a**) Averaged spectra of the analyzed region (600–3000 cm^−1^) for each cell type. SDs are shown by the shaded deviation. (naïve cells: black, activated cells: red, unknown cell: green). (**b**) Kernel density distribution diagram of the first and second principal component axes for each cell type. On the x- and y-axes, each cell type distribution density for PC1 or PC2 is shown as lines. (**c**) The first two loading vectors calculated by PCA. (**d**) Kernel density distribution diagram of the second and third PLS score for training data sets of each cell type. Naïve cells are shown as black lines, while activated cells are shown as red lines. On the x- and y-axes, the cell distribution density for PLS score2 or PLS score3 is shown as lines. Unknown cells data as test data is plotted above the kernel density distribution line of the training data (cells expected to be naïve by PLS-DA: blue, cells expected to be activated cells: orange). Each dot represents a single cell. (**e**) Flow cytometry analysis shows the rate of anti-CD69 positive cells in naïve cells and the unknown state of cells after CD3/CD28-stimulation. The x-axis in each flow cytometry plot indicates fluorescent intensity. The y-axis indicates the number of cells.

To know the cell state of the unknown cells without staining, we measured the Raman spectrum of 5 μm diameter circular regions from 300 unknown cells and compared these with the spectra of activated and naïve cells. (Fig. 6a). Then, we used PCA to extract the features that contributed to the variance of each cell, and performed dimensionality reduction (Supplementary Fig. S6). The cell distribution against PC1 and PC2 axes predicted that the unknown cells were within the same distribution region as the activated and naïve cells, indicating that these cells were either activated or naïve (Fig. 6b,c).

Next, we hypothesized that machine learning could predict the cell state (activated or naïve) of the unknown cells. We used PLS-DA, which allows visualization among supervised learning methods, to discriminate the activation states of the unknown cells. First, we used the spectral data of all activated and naïve cells as training data to create a classification axis (Supplementary Fig. S7). Among the five components extracted by PLS-DA, the distribution of activated and naïve cells against the PLS scores 2 and 3 was the most visually distinct. Subsequently, as test data, unknown cells were classified against the extracted axes, revealing that 41% of the tested unknown cells were expected to be activated cells, and 59% were expected to be naïve cells. Activated and naïve cells used in the training data were represented by kernel density estimation, and unknown cells used in the test data were plotted on the line (Fig. 6d). Other machine learning methods were used to similarly predict the activation states of the unknown cells (Table 3). LDA, SVM, and MLP predicted activation rates of 42, 42, and 38%, respectively.

**Table 3:** Activation ratio by classifier using supervised machine learning (linear discriminant analysis (LDA), partial least squares discriminant analysis (PLS-DA), support vector machine (SVM), and neural network (multilayer perceptron, MLP)).

Method	Ratio (%)	
	Naïve	Activated
LDA	58	42
PLS-DA	59	41
SVM	58	42
MLP	62	38

To verify the accuracy of the values predicted by PLS-DA, we also verified the activation rates of the unknown cells using flow cytometry (Fig. 6e), which indicated that 46.6% of the unknown cells were positive for CD69. Therefore, the predictive accuracy of the activation state by PRESS was roughly consistent with the positive rate of the activation marker CD69, as determined by flow cytometry.

## Discussion

PRESS provides rapid spectral data that can be used for machine learning, enabling highly accurate cell classification without staining. Recently, several methods for high-sensitivity Raman spectroscopy have been reported, including a method of enhancing the weakly scattered light by focusing and irradiating the sample using a two-wavelength laser (Coherent anti-Stokes Raman Scattering: CARS^42^), and another method of enhancing the Raman signal using metal nanocolloids (Surface Enhanced Raman Scattering: SERS^43–45^). Most of these methods are used to produce Raman images and generate a large amount of spectral data, which requires relatively long measurement time and labor-intensive analysis. Conversely, PRESS can acquire averaged spectra over a wide area in a single scan, thereby reducing site-associated variation in measurements and avoiding the need for data processing, such as measuring multiple points on a single cell and averaging their spectra. Using PRESS reduces the exposure time per measurement area, thus reducing photothermal damage to the cell. In addition, compared to methods that use a low NA objective lens to irradiate the laser over a wide area^21,22^, PRESS can quickly acquire a Raman spectrum from a wide area with high sensitivity, as the laser irradiates in a swivel pattern and moves around a specific area. Unlike the random scan system^23^, the PRESS enables irradiation with the laser beam uniformly over a wide area, with controlled scanning speed and frequency. Nevertheless, there is a limit to the measurement area, which depends on the magnification of the objective lens. The larger the measurement area, the longer the exposure time and the higher the probability of detecting cosmic rays. While we measured circular regions for cells in suspension, the PRESS can also obtain spectra in a square region. When measuring cells of varying shapes, such as adherent cells, a system is required to pre-measure the shape of the cell and reflect it in the scan area, for e.g., by using a quantitative phase contrast microscope^46,47^.

T cell activation is induced in a step-by-step process. At 4–6 h after stimulation, interleukin-2 (IL2) transcription and secretion are stimulated, followed by the expression of CD69, the IL-2 receptor CD25, CD40L, and HLA-DA. The distribution of cells, as shown by PLS-DA, indicates that there is variation within activated T cells. To use machine learning to assess the activation stage, we needed to prepare training data of T cells sorted by surface markers of subdivided activated T cells. By learning the spectrum of progressively activated T cells, detailed discrimination can be performed non-invasively during the activation phase of T cells. Alternatively, other machine learning algorithms (unsupervised machine learning methods or clustering) can be used to extract cell transitions and characteristic peaks in the activation phase, or to discover unknown cells in the process.

The obtained spectra were analyzed using machine learning. In the field of analytical chemistry and photonics, machine learning has been extensively used as a method to extract characteristic information from complex and large data sets. For example, in the analysis of cell-derived spectra, unsupervised learning methods such as PCA, and supervised learning methods such as SVM, have been successfully used to classify cells^4–7^. Samples with large spectral variability can be clearly classified by unsupervised learning methods (Fig. 3j,k), whereas supervised learning methods are more effective for samples with small spectral variability (Fig. 5d–f). In the present study, characteristics of samples that could not be differentiated by PCA, such as activation state, were clearly distinguished by PLS-DA and SVM. However, supervised learning methods cannot be used without class-labeled data, which is a major disadvantage. In addition, with insufficient samples, the classification accuracy decreases. The required number of samples depends on the extent of change in sample characteristics and the variation within the classes. Although we were able to classify four different cell types with high accuracy using 50 spectra each, it was necessary to perform a classification using more spectral data of each cell type, such as early or late activation state, for a more detailed classification. The required amount of data for classification needs to be adjusted accordingly based on the spectral variation and classification accuracy.

In addition to the requirement for the amount of data to be measured, the state of the cell must also be carefully controlled. We measured cells under two conditions: adhered to a quartz glass dish and as single cell suspensions. In both conditions, PCA distributions overlapped, but LDA was able to clearly classify different cell types and states. A comparison between cells in suspension and adherent cells indicated that the cellular components differed greatly, depending on the morphological changes to the cells. PRESS is sensitive enough to be able to distinguish between adherent and suspended cells, even if the cells are derived from the same culture conditions. Therefore, cells in the same physical state must be examined to verify the homogeneity of the cells.

In the present study, we showed that the PRESS with supervised machine learning can quickly acquire Raman spectra from a wide area in a single cell and reduce site-dependent variations in measurement without damaging the cells. It also enables us to classify multiple cell types and different cell states within the same cell noninvasively. This highly accurate and non-staining cell sorting method has many potential applications in basic scientific research, such as the identification of unclassified novel cell types and states, and may also contribute to the development of low-cost and highly accurate drug screening methods.

## Methods

### Cells

All procedures were performed in accordance with the guidelines of the Committee for the Ethics on Experiments with Human Derivative Samples of the National Institute of Advanced Industrial Science and Technology (AIST) (Approval Number: 2014-169). The experiments involving the use of human induced pluripotent stem cell (iPSC) were also approved by the Ethics Committee of AIST.

Mouse embryonic fibroblasts (MEFs, Cat. M-FB-481, Lonza, Basel, Switzerland) were grown on 0.1 w/v% gelatin solution (FUJIFILM Wako Pure Chemical Corporation, Osaka, Japan)-coated culture dishes, and cultured in Dulbecco’s modified Eagle’s medium (DMEM) (FUJIFILM Wako Pure Chemical Corporation, Osaka, Japan) supplemented with 10% fetal bovine serum (FBS; Biowest, Nuaillé, France), 1% Glutamax (× 100, Invitrogen, Carlsbad, CA), and 1% penicillin–streptomycin solution (× 100; FUJIFILM Wako Pure Chemical Corporation, Osaka, Japan).

Immortalized human mesenchymal stem cells (hMSCs) (SCRC-4000, ATCC, Manassas, Virginia, USA) were cultured in DMEM supplemented with 10% FBS and 1% penicillin–streptomycin solution (× 100). When MEFs or hMSCs approached confluence, the cells were digested into single cells using TrypLE Express Enzyme (× 1, Thermo Fisher Scientific, Waltham, MA, USA) and the resulting cells were passaged or used for Raman spectroscopy.

Human iPSCs (hiPSCs) (201B7 line, female, RIKEN BRC, Tsukuba, Japan) were maintained in mTeSR1-cGMP feeder-free maintenance medium (Stemcell Technologies, Vancouver, Canada) on Laminin511-E8 (iMatrix511; Nippi, Tokyo, Japan)-coated culture plates. The culture medium was changed daily. When hiPSCs approached confluence, the colonies were digested into single cells using Accutase (Thermo Fisher Scientific, Waltham, MA, USA), and the cells were passaged or used for Raman spectroscopy.

Jurkat cells (human peripheral blood T lymphocyte) were maintained in RPMI-1640 medium (FUJIFILM Wako Pure Chemical Corporation, Osaka, Japan) supplemented with 10% FBS and 1% penicillin–streptomycin solution (× 100). Jurkat cells were provided by Dr. Ryoji Yagi (Chiba University, Japan). All cells were grown in an incubator at 37 °C under 5% CO_2_ in a humidified atmosphere.

### T cell activation

For Raman measurement, Jurkat cells were passaged at a density of 5 × 10^5^ cells mL^−1^ in culture medium and stimulated by Dynabeads CD3/CD28 T Cell Expander (Thermo Fisher Scientific, Waltham, MA, USA) to obtain a bead-to-cell ratio of 1:1 for 24 h. To assess the uptake of free fatty acids, Jurkat cells were cultured in 24-well plates pre-coated with anti-CD3 (5 μg mL^−1^) and soluble anti-CD28 (1 μg mL^−1^) antibody (BioLegend, CA, USA) for 24 h. Cells were stimulated in an incubator at 37 °C under 5% CO_2_ in a humidified atmosphere.

### Flow cytometry

Cells were stained with fluorescein (FITC)-conjugated anti-human CD69 antibody (BioLegend, CA, USA) in D-PBS (-) (FUJIFILM Wako Pure Chemical Corporation, Osaka, Japan ) containing 1% BSA (FUJIFILM Wako Pure Chemical Corporation, Osaka, Japan) and 5 mM EDTA (DOJINDO laboratory, Kumamoto, Japan) on ice for 15–20 min in the dark. Fluorescence intensity was detected with BD FACSVerse (BD Biosciences, New Jersey, USA). The flow cytometry data were analyzed using FlowJo v10.7 software^48^ (BD Biosciences, New Jersey, USA).

### Uptake of labelled palmitic acid by activation

Cell were stained with fluorescently labelled palmitate (Bodipy FLC16, 1 μM; Invitrogen, Carlsbad, CA) for 30 min in an incubator at 37 °C under 5% CO_2_ in a humidified atmosphere. After incubation, cells were washed with D-PBS (-) and the fluorescence intensity and positive ratio was analyzed by flow cytometry.

### Quantitative reverse transcription polymerase chain reaction (qRT-PCR) analysis

Total RNA was isolated using NucleoSpin RNA (Macherey Nagel GmbH & Co. KG, Duren, Germany). The purity and concentration of RNA were determined using a NanoDrop Lite spectrophotometer (Thermo Fisher Scientific, Waltham, MA, USA). One microgram total RNA was reverse transcribed to cDNA using the ReverTra Ace qPCR RT Kit (TOYOBO, Osaka, Japan). qRT-PCR was then performed using the LightCycler 96 System (Roche, Basel, Switzerland) with THUNDERBIRD SYBR qPCR Mix (TOYOBO, Osaka, Japan). qPCR data were analyzed using the ddCt method^49^ with *36b4* (RPLP0) used as a housekeeping gene, and are shown as the mean ± standard deviation (SD) of triplicate measurements. The primer sequences used are as follows: *IL-2*, forward 5′-TCAAACCTCTGGAGGAAGTGC-3′, reverse 5′-CATGAATGTTGTTTCAGATCCCTTT-3′, *TNF-*α, forward 5′-CCCATGTTGTAGCAAACCCTC-3′, reverse 5′-TATCTCTCAGCTCCACGCCA-3′, *36B4*, forward 5′-AGATGCAGCAGATCCGCA-3′, reverse 5′-GTTCTTGCCCATCAGCACC-3′.

### Raman microscopy

Raman spectra of cells were obtained with a custom-built Raman microscope based on the Raman system (Confocal Raman Spectrometer STR series; AIRIX corp., Tokyo, Japan), as described previously^23^. The Raman system consisted of a Nikon Ti2-U microscope (Nikon, Tokyo, Japan). A continuous-wave Diode-Pumped Solid-State (DPSS) green 532 nm laser (DL 532-100, maximum power 120 mW) coupled with an optical fiber, was used for excitation. A Lambda 40 × C/NA 0.95 objective lens (Nikon, CFI Plan Apo Lambda) was used at approximately 40 mW power in the sample stage, producing a laser spot size of < 460 nm. A laser spot size is calculated by the following formula: Spot size = (2 × M2 × wavelength)/(π × NA). M2 is the beam quality, which in our system is 1.1, wavelength is 532 nm, and NA is 0.9. As a result, the spot size is expected to be 414 nm. In fact, when using an objective lens with 100 × objective lens (NA 0.9), the real laser spot size was measured to be 455.4 nm. Backscattered Raman signals were dispersed by a spectrometer (STR200-2LC, AIRIX) equipped with 1200 g grating and detected with a CCD camera (iVac316, Andor Technology).

To build the PRESS for measuring a wide area, two biaxial galvano mirrors were placed in the laser optical path (Fig. 1a). The 532 nm DPSS laser light enters the optical path through a laser optical fiber and passes through a neutral density filter (NDF) and a bandpass filter (BPF). The laser is reflected by a flat mirror and a dichroic mirror and then arrives at the biaxial galvano mirrors. By rotating the galvano mirrors at high speed, the laser light can be irradiated to a specific area of the sample in a swirling pattern (Fig. 1b–f, Movie 1, Supplementary Fig S1a). The scattered light from the sample passes through the dichroic mirrors again, via the same path as the laser incidence path. The scattered light is further transmitted through a high pass filter (HPF) to allow only the high frequency components to pass through, so that only the Raman scattering light is focused on the spectrometer through the optical fiber. The Raman scattering light is detected by a CCD detector as a spectrum, with the Raman shift (wavenumber, cm^−1^) plotted on the horizontal axis and the intensity of the scattered light (a.u.) on the vertical axis. The detectable Raman spectrum region was 46–3110 cm^−1^.

By controlling the rotational speed of the galvano mirrors, the number of exposures in the area can be changed. The marking speed of the laser was 0.002–2 mm ms^−1^. The area to be irradiated can be specified as a circle (0.5–300 μm diameter) or a square (0.5–300 μm X, Y distance). In the present study, a circular region was measured to specifically classify cells in suspension.

### Raman data acquisition

For calibrating the spectrometer, we measured the spectra of Raman shift standards and sulfur before sample measurement. The spectrum of sulfur was detected by point-scan system with an exposure time of 1 s using NDF 1 or 5%. Five characteristic spectral peaks were detected (50.0, 85.1, 153.8, 219.1 and 473.2 cm^−1^), and calibration was performed with three points (153.8, 219.1, and 473.2 cm ^−1^) (ASTM E1840-96, 2014, https://doi.org/10.1520/E1840-96).

To validate the function of the PRESS, we measured the Raman spectra of solid palmitic acid (FUJIFILM Wako Pure Chemical Corporation, Osaka, Japan), which is frequently used in biochemistry and has known Raman peaks. Palmitic acid crystals were measured on quartz glass culture dish with a diameter of 35 mm (BMS, Tokyo, Japan). The measurements were conducted by point-scan or PRESS in circular areas with diameters 5, 10, and 20 μm. The exposure time was 3 s, and the laser marking speed was 1 mm ms^−1^. To confirm that the peaks detected belonged to palmitic acid, we used the spectral database KnowItAll^26^ (John Wiley & Sons, Inc., NY, USA. A palmitic acid code: FFRX #478).

To evaluate the PRESS for classification of cell types, the Raman spectra of Jurkat, MEFs, hMSCs, and hiPSCs in suspension or adhered on quartz glass dishes were measured. The cells were not fixed, and measurements were performed on live cells. For adhered cells, the cells were washed twice with D-PBS (-) and measured in D-PBS (-). The Raman spectra were obtained by irradiating a circular area of 10 μm diameter. A hundred cells were randomly selected from each cell type and measured. For cells in suspension, the cells were dispersed into single cells with TrypLE or Accutase, washed twice with D-PBS (-), and measured in D-PBS (-) on a quartz glass dish. The Raman spectra were obtained by irradiating a circular area of 10 μm diameter with a laser. Fifty cells were randomly selected from each cell type and measured. For classification of the activation state of Jurkat cells, Raman spectra were obtained by irradiating a circular area of 5 μm diameter with a laser. The spectral data were obtained from 300 activated and naïve cells each over 3 days.

For all cell measurements, the duration of exposure to the laser was 3 s, and the laser marking speed was 0.5 mm ms^−1^. This oscillation rate allowed the laser light to move back and forth more than once in the region during the exposure time. The measurement area (5 or 10 μm circle in diameter) was specified to include 50% of the nuclear and 50% of the cytoplasmic regions of the cell, to reduce the variability of the measurement area.

### Raman spectrum analysis

The Raman spectra were analyzed using Python 3.7.7 (Continuum Analytics, Inc., Newport Beach, CA, USA). Pre-processing of data was performed as previously reported^3^. Briefly, the spectra were first smoothed using the Savitzky–Golay function (second polynomial order). Second, the baseline was corrected using a polynomial function (order 4) and vector normalized to the 600–2980 cm^−1^ spectral range. To detect characteristic peaks in the spectral data, we first calculated the absolute value of the change (difference) between the average spectrum of naïve cells and the average spectrum of activated cells at each wavenumber (600–2980 cm^−1^) (Fig. 4d). We then searched for peaks using Python's scipy.signal, setting a threshold value (> 3.5% change). Among the detected peaks, we extracted 8 peaks that were significantly different between samples (Student *t* test or Welch *t* test, *P* < 0.05).

### Machine learning classification

To reduce the dimensionality of the Raman spectrum, principal component analysis (PCA) of the data was conducted to extract the principal component (PC) loading vectors and PC scores. The first five PCs were selected as they accounted for majority of the variance in the dataset.

Next, we analyzed the performances of four-well known parameters in machine learning to classify three or four cell types, and to discriminate between different activation states using the obtained Raman spectra. For the supervised learning analysis, we used smoothed, baseline corrected, and vector normalized data before dimensionality reduction by PCA. We applied linear discriminant analysis (LDA), partial least squares discriminant analysis (PLS-DA), support vector machine (SVM), and a neural network (multilayer perceptron, MLP). For classification accuracy, we performed cross validation. All spectral data were mixed and separated randomly into 80% training data and 20% test data. When we investigated the activation state of unknown cells, all data of activated and naïve cells (600 spectra) were used as the training dataset, and the data of unknown cells (300 spectra) was used as the test dataset. All data were processed in Python. PCA, and all classifications were analyzed using the Scikit-learn Python package.

We comprehensively evaluated the models based on ROC curves and ten-fold cross validation. In the process of generating the ROC curves, performance parameters for all models were calculated based on the sensitivity and specificity of each class, as well as the overall accuracy rate according to:

$${\text{Sensitivity }} = {\text{ TP}}/\left( {{\text{TP}} + {\text{FN}}} \right), \, \;{\text{Specificity }} = {\text{ TN}}/ \, \left( {{\text{TN}} + {\text{ FP}}} \right),\;{\text{ and }}\;{\text{Accuracy }} = \, \left( {{\text{TP}} + {\text{TN}}} \right)/\left( {{\text{TP}} + {\text{TN}} + {\text{FP}} + {\text{FN}}} \right),$$

where TP is the number of true positives, FP is the number of false positives, TN is the number of true negatives, and FN is the number of false negatives. Using these values, we plotted the ROC curves. The X-axis represented the false positive rate (FPR, 1-specificity), and the Y-axis indicated the true positive rate (TFR). The area under the curve (AUC) was calculated to determine the accuracy of the model. Additionally, the root mean square error (RMSE) was evaluated.

RMSE is one of the indicators to evaluate the error of the regression model. RMSE therefore indicates the error value between the original label ${y}_{i}$ (i = 0 or 1), naïve cells is “0” and activated cells is “1”) and the label predicted from the classification model (($\widehat{{y }_{i}}$) (I = 0 or 1), naïve cells is “0” and activated cells is “1”), and is calculated by the following formula:

$$RMSE=\sqrt{\frac{1}{n}\sum_{i=1}^{n}{\left({y}_{i}-\widehat{{y}_{i}}\right)}^{2}}$$

To extract the five axes contributing to the classification in PLS-DA, five PLS scores were allocated to each cell in the training and test datasets. This complicated the evaluation of the sensitivity and accuracy of the binomial classification. Therefore, using the Mahalanobis' distance, the five scores were changed to values for binomial classification. First, we calculated the central points of the two classes (activated or naïve cells) created by the training data. Then, we calculated the Mahalanobis' distance between the central points of the two classes and the test data and classified them as a class with a lower Mahalanobis' distance value. Consequently, the value of each test data from the five PLS-DA scores was changed to a binomial value. Thus, the sensitivity, specificity, and accuracy could be obtained by reducing the dimensionality to five dimensions through PLS-DA and classifying the data into two classes based on the Mahalanobis' distance.

### Statistics and reproducibility

All data are expressed as mean ± SD values. Differences between experimental groups were analyzed by Student’s *t* test or Welch’s *t* test (two groups). Differences among more than two groups were analyzed via one-way ANOVA and Tukey’s post hoc method was used for multiple comparisons. Differences with *P* < 0.05 were considered statistically significant. Symbols used are: *P < 0.05 and n.s. (not significant) *P*≧0.05.

## Supplementary Information

Supplementary Information 1.

Supplementary Video 1.

Supplementary Information 2.

## Data Availability

The data that support the findings of this study are available from the corresponding authors upon reasonable request.

## Code availability

The software codes used for analyzing the data are available from the corresponding authors upon reasonable request.
