# Supplementary material for: Non-invasive cell classification using the Paint Raman Express Spectroscopy System (PRESS)
Source: Sci Rep. 2021 Apr 23;11:8818. doi: 10.1038/s41598-021-88056-3 (PMC8065115; doi:10.1038/s41598-021-88056-3)
Supplement: Supplementary file 1 — Supplementary Information 1. [file 41598_2021_88056_MOESM1_ESM.docx]

**Description of Additional Supplementary Files**

**Supplementary Movie 1:**

**PRESS: A system to acquire a high-sensitivity Raman spectrum from a wide area in a single measurement.** Video of laser scanning in a circular area of 20 μm diameter. By using a galvano mirror, the laser scans the circular area in about 3 seconds.
