# Supplementary material for: Non-invasive cell classification using the Paint Raman Express Spectroscopy System (PRESS)
Source: Sci Rep. 2021 Apr 23;11:8818. doi: 10.1038/s41598-021-88056-3 (PMC8065115; doi:10.1038/s41598-021-88056-3)
Supplement: Supplementary file 3 — Supplementary Information 2. [file 41598_2021_88056_MOESM3_ESM.docx]

**Supplementary Figure.**

Title: **Non-invasive cell classification using the Paint Raman Express Spectroscopy System (PRESS)**

Yuka Akagi^1,2,3^, Nobuhito Mori^1^, Teruhisa Kawamura^4^, Yuzo Takayama^1^, Yasuyuki S. Kida^1,2, 5*^

^1^ Cellular and Molecular Biotechnology Research Institute, National Institute of Advanced Industrial Science and Technology (AIST), Central 5-41, 1-1-1 Higashi, Tsukuba, Ibaraki, 305-8565, Japan

^2^Advanced Photonics and Biosensing Open Innovation Laboratory, National Institute of Advanced Industrial Science and Technology (AIST), Central 5-41, 1-1-1 Higashi, Tsukuba, Ibaraki, 305-8565, Japan.

^3^Tsukuba Life Science Innovation Program (T-LSI), School of Comprehensive Human Sciences, University of Tsukuba, 1-1-1 Tennoudai, Tsukuba, Ibaraki 305-8572, Japan

^4^Department of Biomedical Sciences, College of Life Sciences, Ritsumeikan University, 1-1-1 Noji-higashi, Kusatsu, Shiga 525-8577, Japan

^5^School of Integrative & Global Majors, University of Tsukuba, 1-1-1 Tennoudai, Tsukuba, Ibaraki 305-8572, Japan

## Supplementary Figure. S1


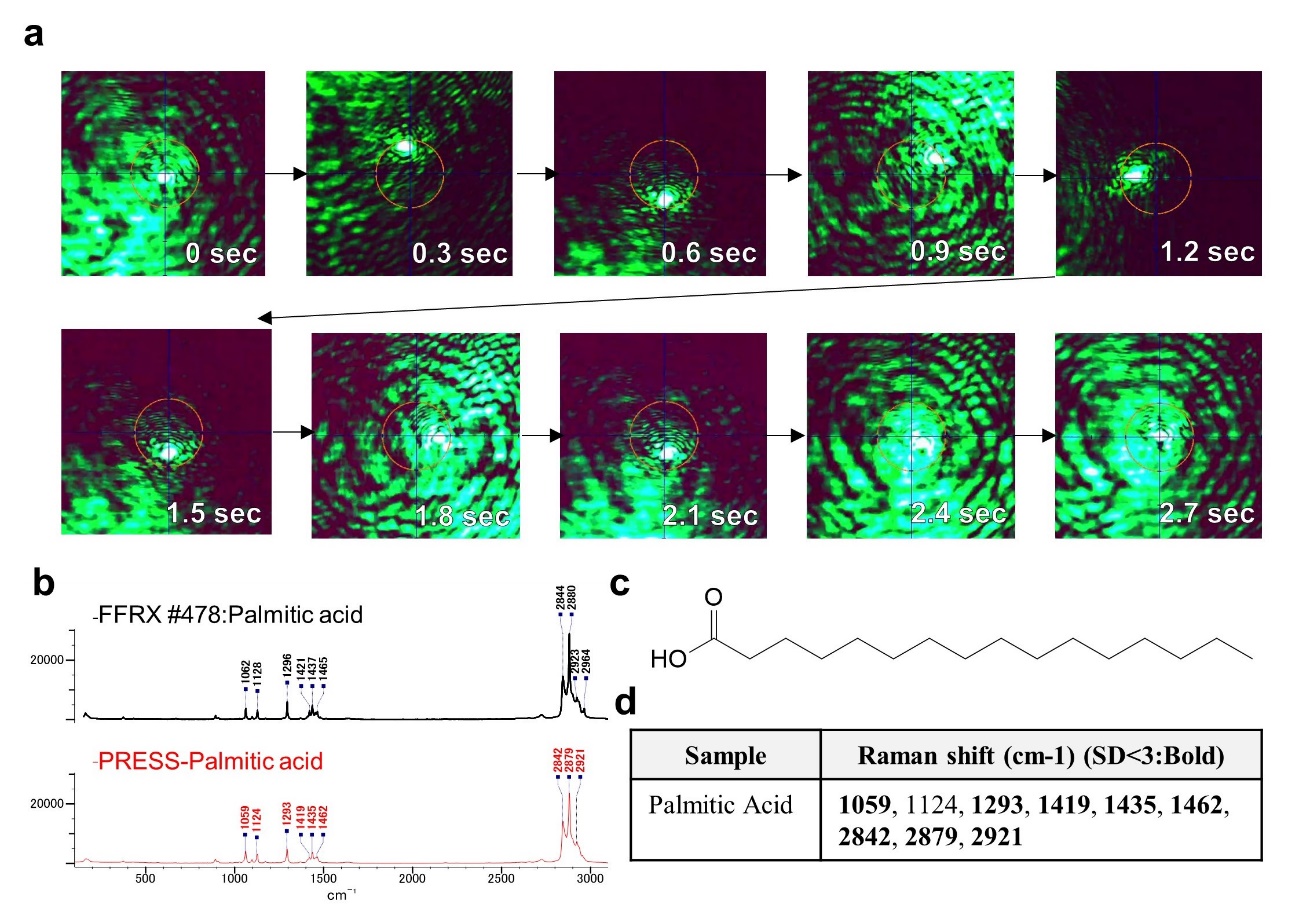


**Supplementary Figure S1. Verification of palmitic acid spectra obtained by the PRESS against a Raman spectral database. a** Time-lapse view of the laser scanning process in a circular area of 20 μm in diameter. A galvano mirror is vibrated at a rate of 1 mm/ms to irradiate the circular region in approximately 3 s. **b** Raman spectra of palmitic acid. Top row: palmitic acid spectra measured by PRESS; Bottom row: palmitic acid spectra provided by spectra database KnowItAll^19^. **c** Chemical structure of palmitic acid. **d** List of detected peak regions. Bold letters indicate peaks with an error of less than 3 between the detected peaks and the database spectrum.

## Supplementary Figure. S2


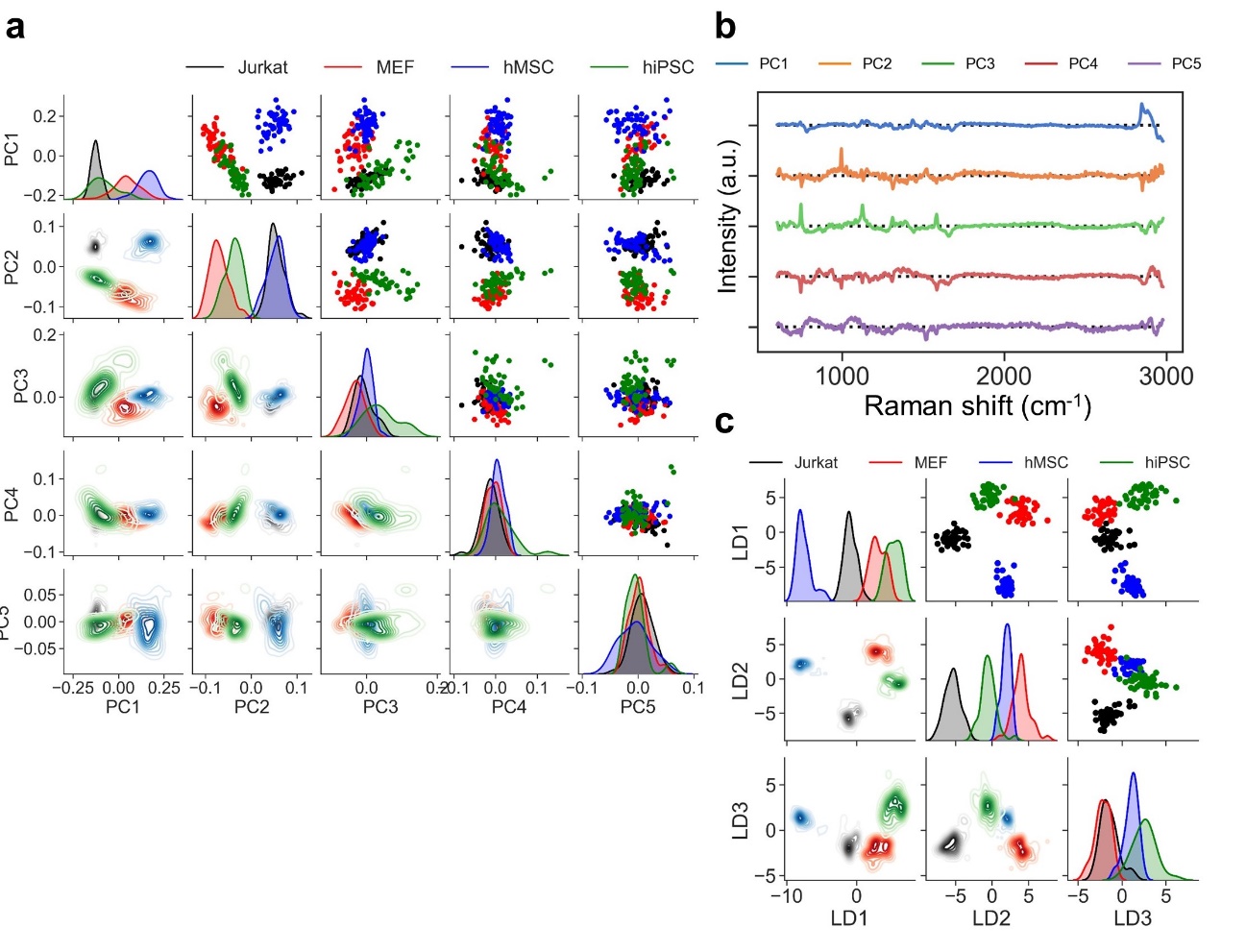


**Supplementary Figure S2. Classification of four typical cell types (Jurkat cells, MEFs, hMSCs, hiPSCs) in suspension by PRESS. a** Pair plot comparing the first five principal components according to PCA, indicating the separability of classes and the distributions for classification. The upper panels show scatter plots where each dot represents a single cell. The lower panels show the Kernel density distribution diagram for each cell type. (Jurkat: black, MEFs: red, hMSCs: blue, hiPSCs: green). **b** The first five loading vectors calculated by PCA. **c** Pair plot comparing the first three LDA scores (LD1~3) according to LDA, indicating the separability of classes and the distributions for classification of cells in suspension. The upper panels show scatter plots where each dot represents a single cell. The lower panels show the Kernel density distribution diagram for each cell type. (Jurkat: black, MEFs: red, hMSCs: blue, hiPSCs: green)

## Supplementary Figure. S3


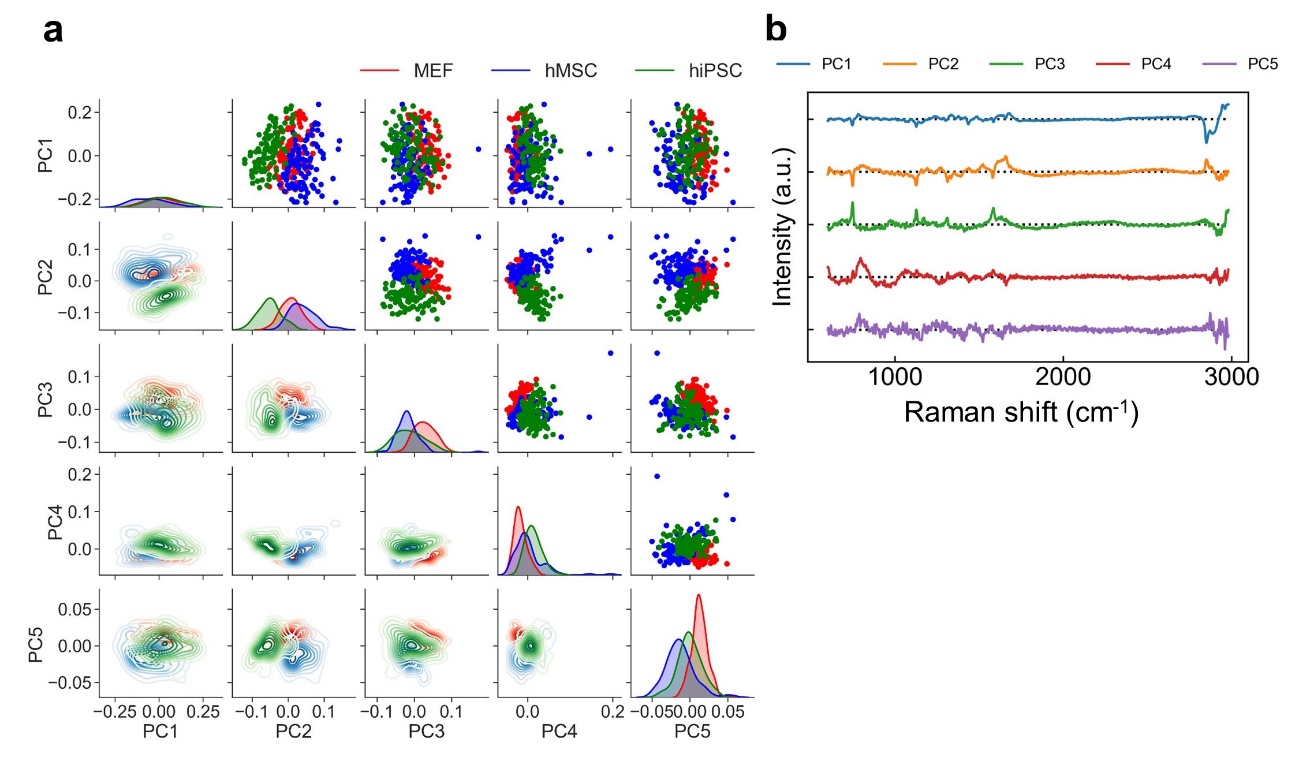


**Supplementary Figure S3. Classification of three typical adhesive cell types (MEFs, hMSCs, hiPSCs) by PRESS. a** Pair plot comparing the first five principal components according to PCA, showing the separability of classes and the distributions for classification. The upper panels show scatter plots where each dot represents a single cell. The lower panels show the Kernel density distribution diagram for each cell type. (MEFs: red, hMSCs: blue, hiPSCs: green). **b** The first five loading vectors calculated by PCA.

## Supplementary Figure. S4


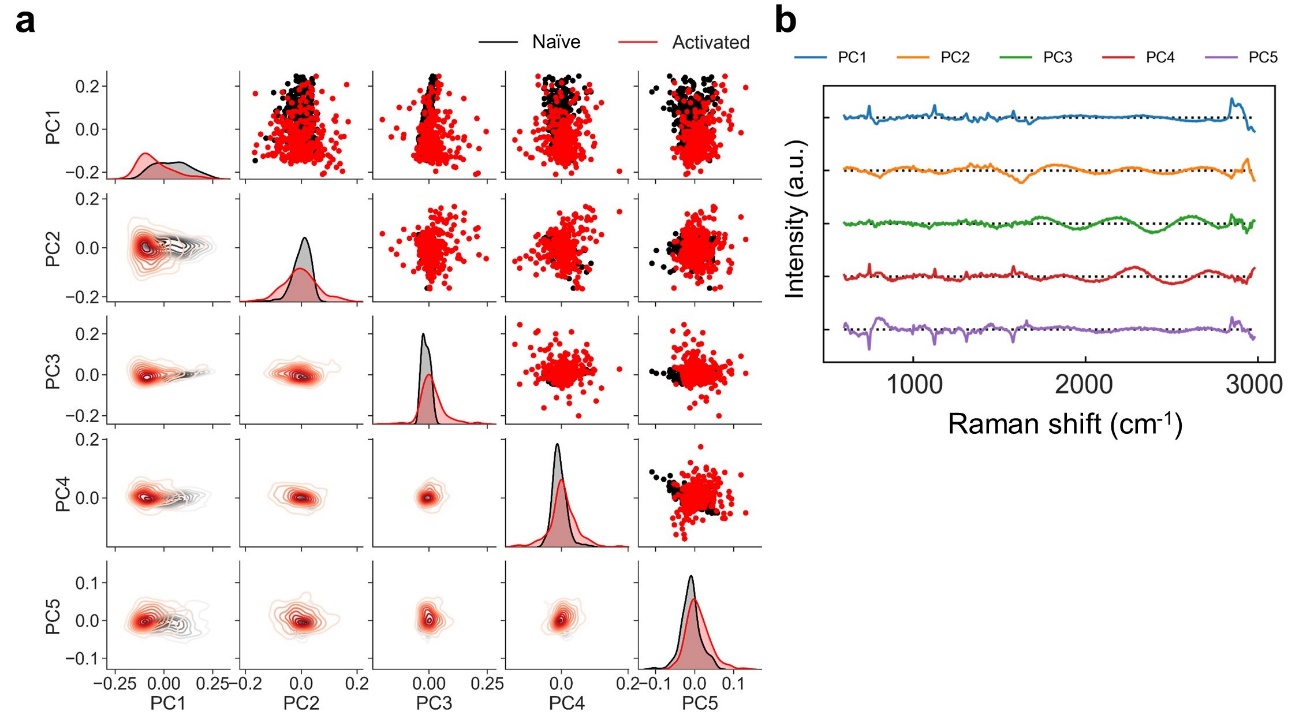


**Supplementary Figure S4. Classification of activated and naïve cells using PCA. a** Pair plot comparing the first five principal components according to PCA, showing the separability of classes and the distributions for classification. The upper panels show scatter plots where each dot represents a single cell. The lower panels show the Kernel density distribution diagram for each cell type (naïve cells: black, activated cell: red). **b** The first five loading vectors calculated by PCA.

## Supplementary Figure. S5


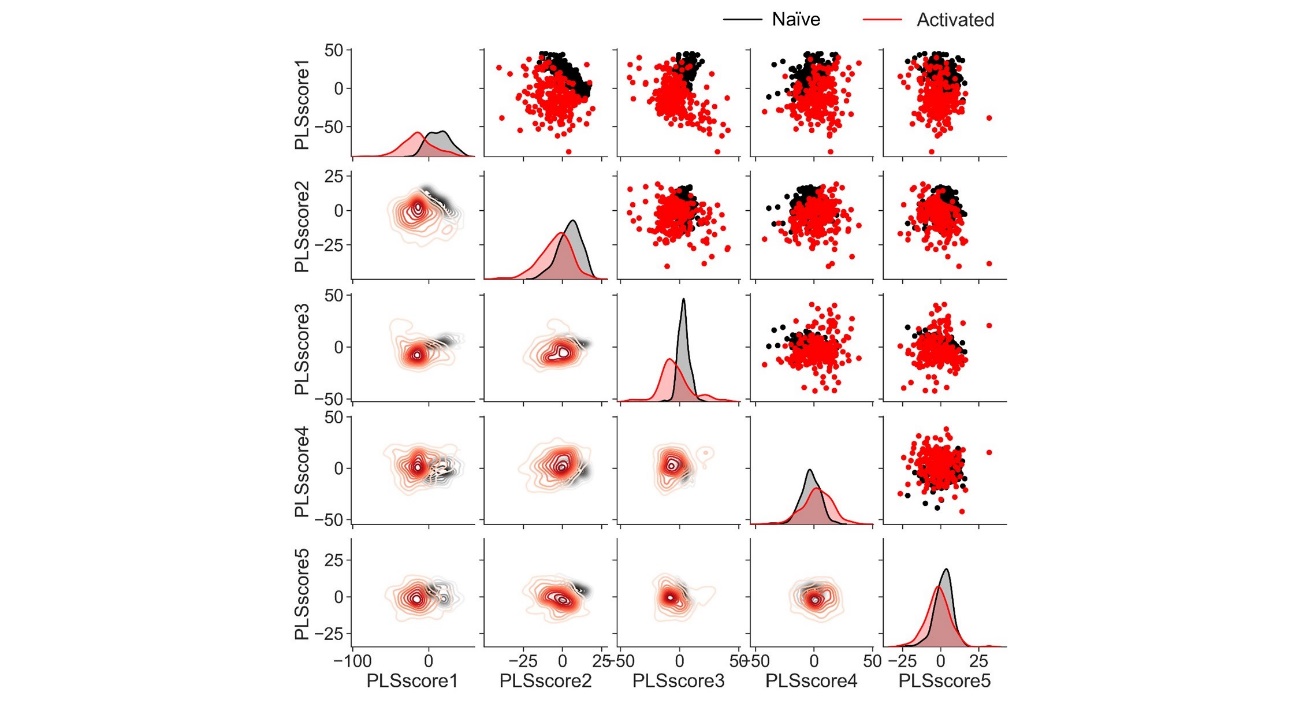


**Supplementary Figure S5. Classification of activated and naïve cells using PLS-DA.** Pair plot comparing the first five PLS scores according to PLS-DA, showing the separability of classes and the distributions for classification. The upper panels show scatter plots where each dot represents a single cell. The lower panels shows Kernel density distribution diagrams for each cell type (naïve cell: black, activated cell: red).

## Supplementary Figure. S6


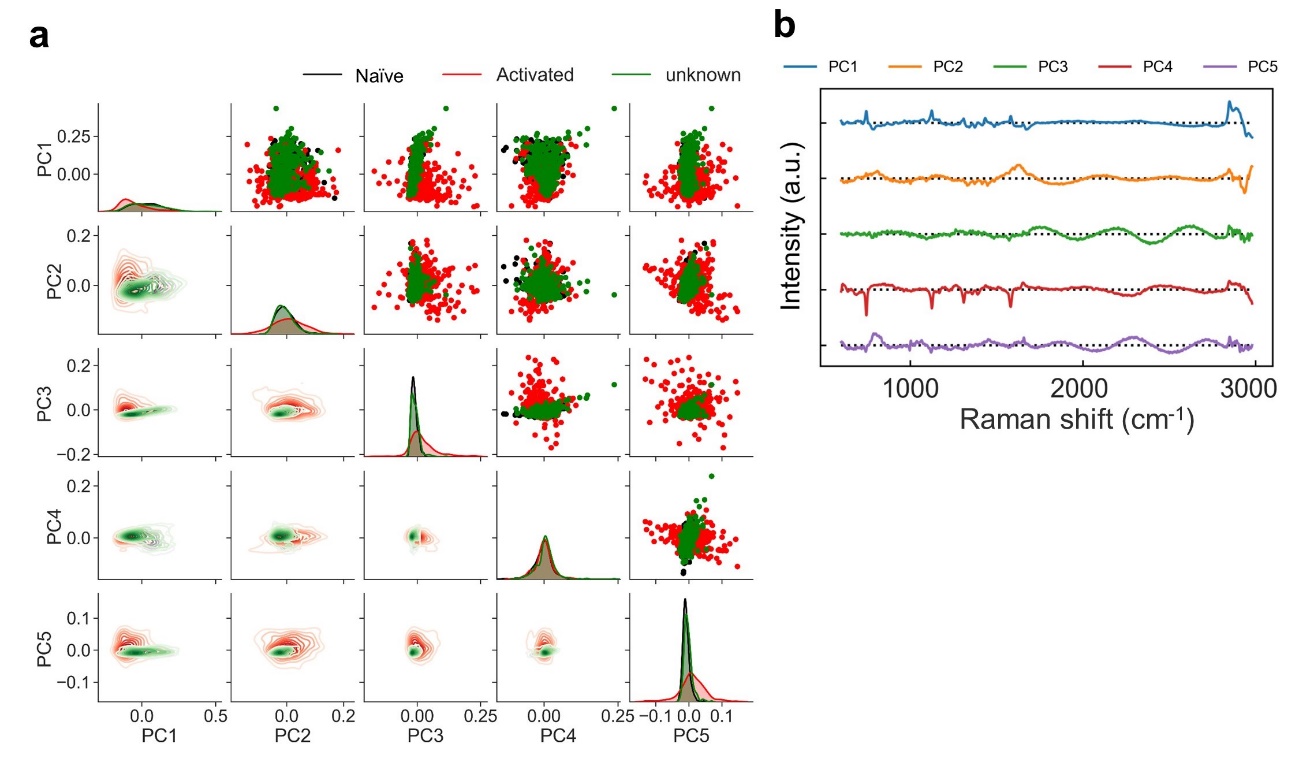


**Supplementary Figure S6. Scatter diagram of unknown state Jurkat cell using PCA.** **a** Pair plot comparing the first five principal components created by PCA between each other, showing separability of classes and distributions for classification. Upper panel shows scatter plot, and each dot represents a single cell. Lower panel shows Kernel density distribution diagram per each cell type. (Naïve cell: black, activated cell: red, unknown cells: green). **b** The first five loading vectors calculated by PCA.

## Supplementary Figure. S7


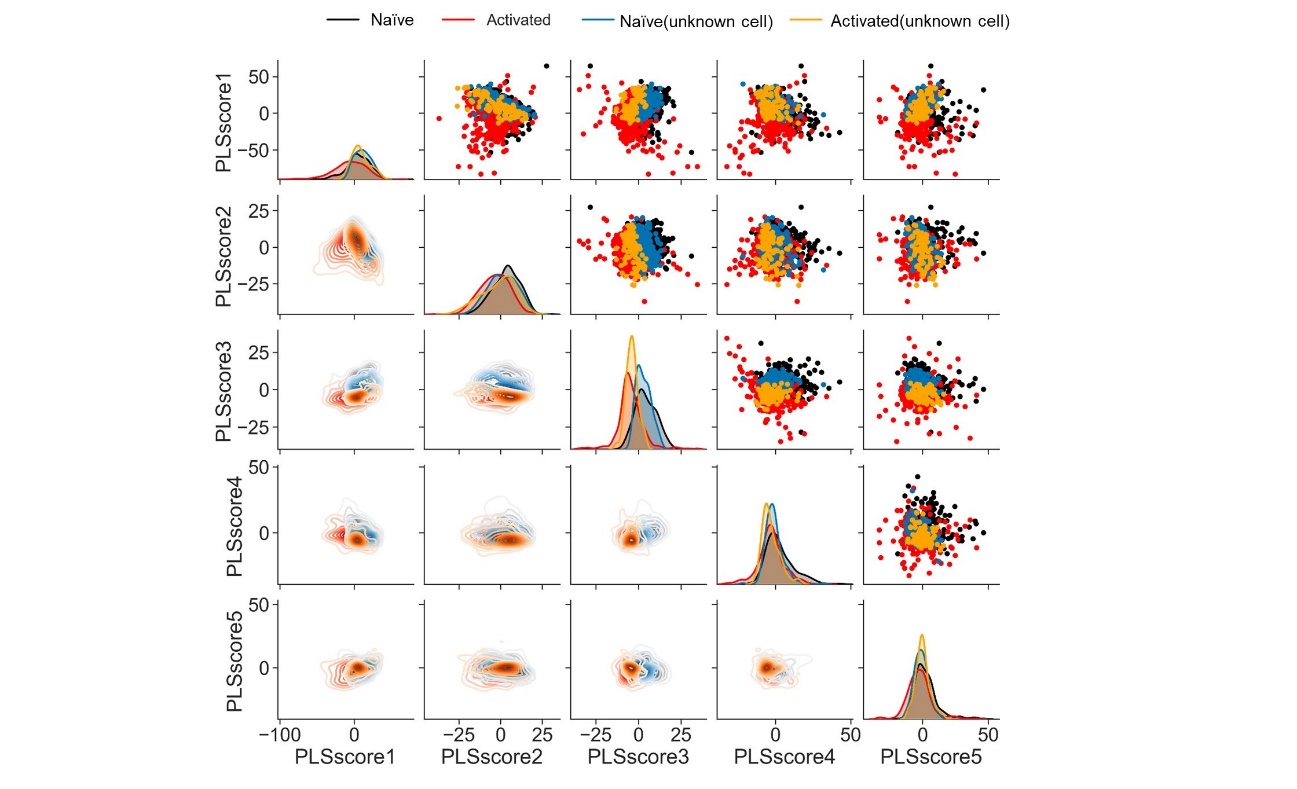


**Supplementary Figure S7. Prediction of unknown state Jurkat cells using PLS-DA.** Pair plot comparing the first five PLS scores created by PLS-DA, showing separability of classes and distributions for classification. Upper panel shows scatter plot, and each dot in represents a single cell. Lower panel shows Kernel density distribution diagram per each cell type. (Naïve cell: black, activated cell: red, Cells expected to be naïve by PLS-DA: blue, cells expected to be activated cells: orange)
